# The Internal Sequence of the Peptide-Substrate Determines Its N-Terminus Trimming by ERAP1

**DOI:** 10.1371/journal.pone.0003658

**Published:** 2008-11-06

**Authors:** Irini Evnouchidou, Frank Momburg, Athanasios Papakyriakou, Angeliki Chroni, Leondios Leondiadis, Shih-Chung Chang, Alfred L. Goldberg, Efstratios Stratikos

**Affiliations:** 1 National Centre for Scientific Research “Demokritos”, IRRP, Aghia Paraskevi, Greece; 2 Department of Molecular Immunology, German Cancer Research Centre, (DKFZ), Heidelberg, Germany; 3 Institute of Physical Chemistry, National Centre for Scientific Research “Demokritos”, Aghia Paraskevi, Greece; 4 National Centre for Scientific Research “Demokritos”, Institute of Biology, Aghia Paraskevi, Greece; 5 Institute of Microbiology and Biochemistry, Department of Biochemical Science and Technology, National Taiwan University, Taipei, Taiwan, Republic of China; 6 Department of Cell Biology, Harvard Medical School, Boston, Massachusetts, United States of America; Cairo University, Egypt

## Abstract

**Background:**

Endoplasmic reticulum aminopeptidase 1 (ERAP1) trims N-terminally extended antigenic peptide precursors down to mature antigenic peptides for presentation by major histocompatibility complex (MHC) class I molecules. ERAP1 has unique properties for an aminopeptidase being able to trim peptides *in vitro* based on their length and the nature of their C-termini.

**Methodology/Principal Findings:**

In an effort to better understand the molecular mechanism that ERAP1 uses to trim peptides, we systematically analyzed the enzyme's substrate preferences using collections of peptide substrates. We discovered strong internal sequence preferences of peptide N-terminus trimming by ERAP1. Preferences were only found for positively charged or hydrophobic residues resulting to trimming rate changes by up to 100 fold for single residue substitutions and more than 40,000 fold for multiple residue substitutions for peptides with identical N-termini. Molecular modelling of ERAP1 revealed a large internal cavity that carries a strong negative electrostatic potential and is large enough to accommodate peptides adjacent to the enzyme's active site. This model can readily account for the strong preference for positively charged side chains.

**Conclusions/Significance:**

To our knowledge no other aminopeptidase has been described to have such strong preferences for internal residues so distal to the N-terminus. Overall, our findings indicate that the internal sequence of the peptide can affect its trimming by ERAP1 as much as the peptide's length and C-terminus. We therefore propose that ERAP1 recognizes the full length of its peptide-substrate and not just the N- and C- termini. It is possible that ERAP1 trimming preferences influence the rate of generation and the composition of antigenic peptides *in vivo*.

## Introduction

Antigenic peptides presented by MHC class I molecules act as a status indicator of the cell's condition and play a pivotal role in the activation of T-lymphocytes versus pathogens like viruses or in pathological conditions like cancer. Recognition of a MHC-peptide complex by the T-cell receptor (TCR) of a cytotoxic T-lymphocyte can lead to the activation of the T-lymphocyte and to target cell lysis [1]–[4]. The antigenic peptides that are loaded on MHC class I molecules are generally derived from intracellular proteins after degradation by an intricate but not deeply understood proteolytic cascade. The first step of this cascade is considered to be the proteasome – a large cytosolic multi-subunit proteolytic complex that is responsible for the degradation of most intracellular proteins and plays crucial roles in the homeostasis and regulation of many cellular processes [5]–[7]. The proteasome generates fragments (peptides) of the protein it degrades that are subjected to further proteolysis in the cytosol [8]–[10]. A small subset of the peptides generated survives the proteolytic activity in the cytosol and is actively transported to the Endoplasmic Reticulum (ER) by a specialized peptide transporter called Transporter Associated with Antigen Processing (TAP) [11]–[13]. In the ER further trimming of the peptides can occur by ER-resident aminopeptidases before the final products are loaded onto nascent MHC class I chains [14]–[19]. The mature MHC-peptide complexes are transported via the secretory pathway to the cell surface for presentation to T-lymphocytes. The proteasome-generated peptides destined for MHC loading generally have the correct C-terminus for MHC binding but carry N-terminal extensions that need to be trimmed away [20]. This trimming is completed in the ER although it may be initiated in the cytosol. A slightly different version of the proteasome, termed the immunoproteasome, exists in immune surveillance cells like dendritic cells, and is up-regulated by immune response modulators such as interferon-γ. The immunoproteasome generates N-terminal extended antigenic peptide precursors more efficiently than the proteasome, something that may help the peptides survive the proteolytic activity of the cytosol and enhance their chances to be transported into the ER [20].

At least one ER-resident aminopeptidase now named ERAP1 (ER-AminoPeptidase 1) or ERAAP (ER aminopeptidase associated with antigen presentation) has been identified to play important roles in the generation of mature antigenic peptides through its action on N-terminally extended antigenic peptide precursors. This aminopeptidase has been previously identified as A-LAP (Adipocyte-derived leucine aminopeptidase), PILS-AP (puromycin-insensitive leucyl-specific aminopeptidase) and ARTS-1 (aminopeptidase regulator of TNFR1 shedding), although its sub-cellular localization and role in the immune system had not been recognized at that point [21]–[23]. ERAP1 is a 100 kDa, monomeric, soluble zinc aminopeptidase that belongs to the M1 family of metallo-peptidases. ERAP1 is induced by interferon-γ and can degrade peptides 9–15 residues long. ERAP1 has been found to greatly affect presentation of specific antigenic peptides tested, as highlighted by cell based antigen presentation assays as well as with the recent construction of an ERAP1^−/−^ transgenic mouse [24]–[27]. In those studies, ERAP1 deletion had complex effects that varied depending on the epitope examined: the presentation of some epitopes was down-regulated whereas the presentation of others was up-regulated; some epitopes remained unaffected. The molecular basis for these effects is currently unclear. Two enzymatic properties of ERAP1 have been recognized thus far that can help scientists understand its role in antigen presentation. First, although human ERAP1 degrades efficiently relatively long peptides (9–15 residues long) its activity seems to dramatically decrease for peptides 8 residues and smaller [15], [16]. Second, human ERAP1 has been shown to degrade a series of model peptides varying on their C-terminus, with different rates for each peptide, demonstrating a preference for hydrophobic amino acids at that position [16]. However, these unique properties of ERAP1 are not enough to sufficiently explain the enzyme's complex role in antigen presentation.

N-terminally extended precursors of antigenic epitopes that are transported into the ER consist of a very large variety of sequences. To investigate the effect of the peptide sequence in ERAP1 trimming we over-expressed the human enzyme in a baculovirus driven insect cell expression system and used the purified recombinant enzyme to screen collections of peptides for degradation of their N-terminal residues by ERAP1. We found that ERAP1 exhibited very strong preferences for specific amino acids in several positions of the peptide substrate, particularly for hydrophobic and positively charged side-chains. We demonstrate trimming rate differences of up to 100-fold for single residue replacements distal from the N-terminus and over 40,000-fold for several replacements at once. To our knowledge no other aminopeptidase has been described to have such strong preferences for residues distal to the N-terminus. Our results suggest a complex molecular recognition between ERAP1 and its peptide-substrate, spanning the full length of the peptide. The possible repercussions of these substrate preferences in the understanding of ERAP1's role in antigen presentation are discussed.

## Materials and Methods

### Peptides

Peptides of the series *X*YWANATRSG [28], T*X*DNKTRAY, TV*X*NKTRAY, TVD*X*NKTAY, TVDA*X*NKTY, TVNKT*X*RAY, TVDNKT*X*AY [11], TVDNKTRY*X*, and TVDNKTRY*X* [29] with *X* being 8–10 different amino acids have been described. Peptides were synthesized by Fmoc chemistry using an Abimed Multiple Synthesizer AMS422 (Abimed, Langen, Germany). The composition was confirmed by mass spectrometry. Peptides used for the alanine scan were purchased by JPT peptide technologies, Berlin, Germany. All other peptides were purchased by GenScript, New Jersey, USA. Peptides were purified by HPLC and were >95% pure.

### Baculovirus construction

Baculovirus carrying cDNA coding for human ERAP1 was constructed according to the instructions of the Bac-to-Bac® Baculovirus Expression System (Invitrogen). Briefly, the pcDNA6/myc-His-ERAP1 [16] plasmid was digested with EcoRI/PmeI and ligated to a previously digested with EcoRI/StuI pFastBac plasmid. The resulting pFastBac plasmid was used to transform competent DH10Bac E.coli. The bacmid product of recombination in the DH10Bac was isolated by standard DNA preparation methodology and used to transfect SF9 insect cells to produce the recombinant baculovirus. The baculovirus was harvested from the cell supernatant and its viral titer determined by plaque assay. Larger amounts of virus were produced by infecting SF9 cell cultures and collecting the supernatant after infection was established.

### Protein expression and purification

Human recombinant ERAP1 was produced in Hi5 insect cells grown in Excel405™ serum free medium, after infection by baculovirus carrying the ERAP1 gene with a C-terminal hexa-His tag. The enzyme was secreted into the cell medium, which was harvested by centrifugation (3000 rpm, 30 min, 4°C, GSA rotor) in a Sorvall centrifuge. The cell supernatant was subjected to 3 rounds of buffer exchange (10 fold dilution each time) using a large scale diafiltration apparatus versus 5 mM phosphate buffer at pH 7 containing 100 mM NaCl. The supernatant was then concentrated and its composition adjusted to 50 mM phosphate (pH 8), 300 mM NaCl, 10 mM imidazole and immediately loaded onto a HiTrap™ chelating column (Qiagen) that was pre-loaded with Ni(II)SO_4_. The column was washed with the same buffer containing 20 mM imidazole and the protein was eluted using a 20 mM to 150 mM imidazole gradient. The resulting peak was collected and dialyzed versus 10 mM HEPES buffer pH 8 and then loaded on a MonoQ™ column (Pharmacia) and eluted with a 20 mM to 500 mM NaCl gradient. The resulting peak exhibiting highest activity was collected and further purified on a S200 size exclusion column (Pharmacia). The purified enzyme was found to have comparable activity and digestive properties to enzyme expressed previously in 293F cells [16] with regard to fluorigenic dipeptide digestion and QLESIINFEKL peptide digestion (data not shown).

### Measurement of enzymatic activity by fluorescent substrate

The aminopeptidase activity of the recombinant produced ERAP1 was followed during the expression and purification steps by the fluorescent signal produced upon digestion of the substrate L-leucine 7-amido-4-methyl coumarin (Sigma-Aldrich). The same assay was used to monitor the stability of the enzyme upon storage and as a calibration assay for the analysis of enzymatic activity by HPLC.

### Measurement of enzymatic activity by analysis of peptide products on reverse-phase HPLC

**Figure 1:**
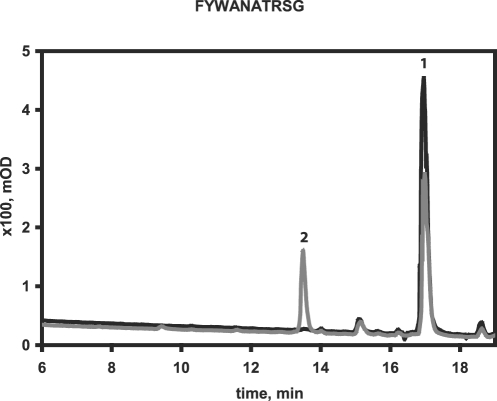
Typical chromatogram of peptide product analysis after ERAP1 digestion. Samples were analyzed by HPLC reverse phase chromatography. 100 µM peptide with sequence FYWANATRSG (written from N-terminus to C-terminus) was mixed with 40 ng of ERAP1 and the mixture was incubated at 37°C. At different time points a sample of the reaction was extracted and mixed with an equal volume of 0.6% Trifluoroacetic acid (to stop the enzymatic reaction) and kept at −20°C until analysis. *Solid line*: Sample at zero time point, indicating the elution of the undigested peptide (peak 1, confirmed by control runs of peptide alone). *Gray line*: Sample after 1 hr of incubation. The surface area of peak 1 is reduced indicating partial digestion of peptide. A new peak can be seen (peak 2) corresponding to the peptide product of the reaction YWANATRSG. The reduction of the surface area of peak 1 is equal to the surface of peak 2 and is typically used to calculate the percent consumption of the peptide substrate.

The digestion of model peptides by ERAP1 was followed by analysis of peptide products of the digestion on a reverse phase C18 column (Higgins Analytical 0546-C183) [16]. Briefly, 100 µM peptide was mixed in 50 µL total volume with 40 to 400 ng purified recombinant enzyme in 20 mM Tris pH 8 buffer containing 100 mM NaCl. The mixture was incubated at 37°C for 30 min to 4 hrs. After incubation the reaction was stopped by the addition of 50 µL of 0.6% TFA and the sample centrifuged for 15 min at 15 000 g. 50 µL of the supernatant were subjected to HPLC analysis. The reverse phase column was equilibrated in either 0.05% trifluoroacetic acid and 5% acetonitrile or 10 mM Sodium Phosphate pH 6.8, 5% acetonitrile before the sample was injected. The elution was done with a 5% to 40% acetonitrile gradient at 1 ml/min, while following the absorbance at 214 nm or 280 nm. Typically, the decrease in peak surface area for a specific peptide (identified by running control experiments or by LC-MS experiments) upon digestion with ERAP1 was used to estimate the amount of peptide that was digested. In all cases, the decrease of the initial peak resulted in the appearance of a new single product peak that had a surface area equal to the surface area decrease of the substrate peak (measured from a control experiment in the absence of enzyme). A typical chromatogram of peptide product analysis after ERAP1 digestion is shown in Figure 1. Several experiments were performed for each peptide tested to fine-tune the reaction conditions (reaction time and amount of enzyme used) and to test reproducibility of results. Each peptide series (varying at one position) was tested in parallel to account for possible variability in the reactions due to changes in enzyme activity upon storage and from preparation to preparation.

### Molecular modelling

Sequence alignment of ERAP1 and Tricorn Interacting Factor F3 (TIFF3) was performed using ClustalW 1.83 [30]. Homology models of ERAP1_[58–948]_ were generated using Modeller 8.2[31] using the 3 crystal structures of TIFF3 (PDB codes: 1Z1W and 1Z5H) as templates. The lowest energy model for each conformation was further subjected to energy minimization in vacuum with AMBER 8 and their stereochemistry was assessed using PROCHECK [32], [33]. Docking of the peptide LMAAFAKAF and LMAAKAKAF in the catalytic cleft of ERAP1 was performed following the procedure described in [34]. Models were analyzed using VMD 1.8.5 and electrostatic potential surfaces were generated using the APBS and PME electrostatics packages [35], [36]. Visualization of the electrostatic potential was performed with PyMol [37].

## Results

### ERAP1 trims the N-terminus of peptides with preference for hydrophobic residues

**Figure 2:**
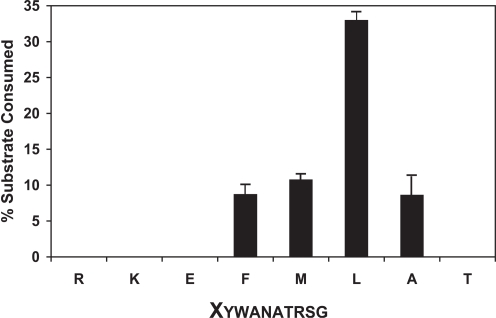
N-terminal specificity of decapeptide trimming by ERAP1. All peptides are based on the same template varying in their N-terminus (indicated as X in the sequence below the graph). 100 µM of each peptide was incubated with 40 ng of ERAP1 at 37°C and the reaction products analyzed as in figure 1. A representative experiment is shown here. Peptides carrying hydrophobic residues at their N-termini were trimmed fastest, whereas peptides carrying charged (R, K or E) or hydrophilic (T) residues in their N-termini were more resistant to cleavage by ERAP1.

ERAP1 has been characterized before as a leucine aminopeptidase because it preferably degrades dipeptide fluorigenic substrates that have a leucine at their N-terminus [22]. However this strong preference conflicts, to a certain extent, with the role of ERAP1 in antigen processing, where it must trim peptides with a vast range of sequences. To address the N-terminal specificity of ERAP1 when degrading peptides we over-expressed human recombinant full-length ERAP1 in insect cell suspension culture and used the purified active enzyme in degradation assays in which we measured the amount of trimming of the N-terminal residue of a panel of peptides that varied only on their N-terminus (Figure 1 and 2). We found that in agreement with the dipeptide digestion results, leucine was the preferred N-terminal residue. Other hydrophobic residues, such as methionine, phenylalanine and alanine were also digested reasonably fast. Non-optimal residues such as charged or hydrophilic in nature, required larger (at least 10 times) amounts of ERAP1 for their removal (data not shown). Overall, ERAP1 appears to indeed act as a leucine aminopeptidase when degrading model peptides although the digestion proceeds, albeit at a much slower rate, for other residues as well.

### Alanine scan of a 10mer peptide template

**Figure 3:**
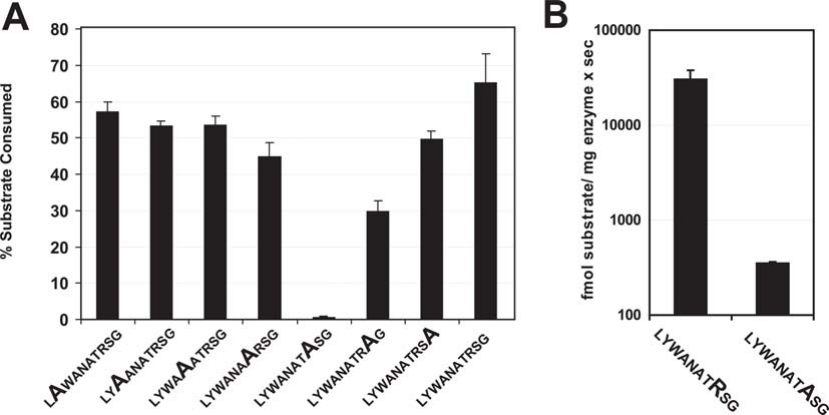
A. Alanine scan of N-terminal trimming of the same 10mer peptide by ERAP1. Peptide variants of the sequence LYWANATRSG designed so that one internal position at a time is sequentially replaced by alanine were analyzed for their susceptibility to N-terminus trimming by 40 ng of ERAP1. Error bars represent the variability between three separate experiments performed in parallel. Less than 1% trimming was detected for peptide LYWANATASG under the conditions of the experiment presented. B. Rates of N-terminus trimming by ERAP1 for peptides LYWANATRSG and LYWANATASG. Substitution of the arginine residue at position 8 by alanine leads to reduction of trimming rates by almost 100 fold. Rates are plotted in logarithmic scale for clarity.

To investigate whether internal residues of the peptide are important for N-terminal trimming by ERAP1 we used a collection of 10mer peptides based on the sequence LYWANATRSG, where one internal residue at a time is replaced by alanine. In every case the removal of the optimal N-terminal residue type (leucine) by ERAP1 was followed by reverse-phase HPLC. Substituting alanine in most positions affected N-terminal trimming by ERAP1 to a moderate degree. Replacement of the arginine residue at position 8 (relative to the N-terminus of the peptide) resulted to a peptide that was surprisingly resistant to N-terminal trimming by ERAP1 (Figure 3A). Specifically, the N-terminus of the peptide LYWANATASG was trimmed with a rate of 0.35±0.02 pmol/µg ERAP1×sec, whereas the control peptide LYWANATRSG was trimmed with a rate 30.4±7.1 pmol/µg ERAP1×sec, a rate almost 100 fold higher (Figure 3B). This finding indicates that internal positions of the peptide substrate can be just as important in determining N-terminal trimming as the nature of the N-terminus.

### Trimming of the N-terminus of a collection of 9mer peptides by ERAP1 is affected by the internal sequence of the peptide

**Figure 4:**
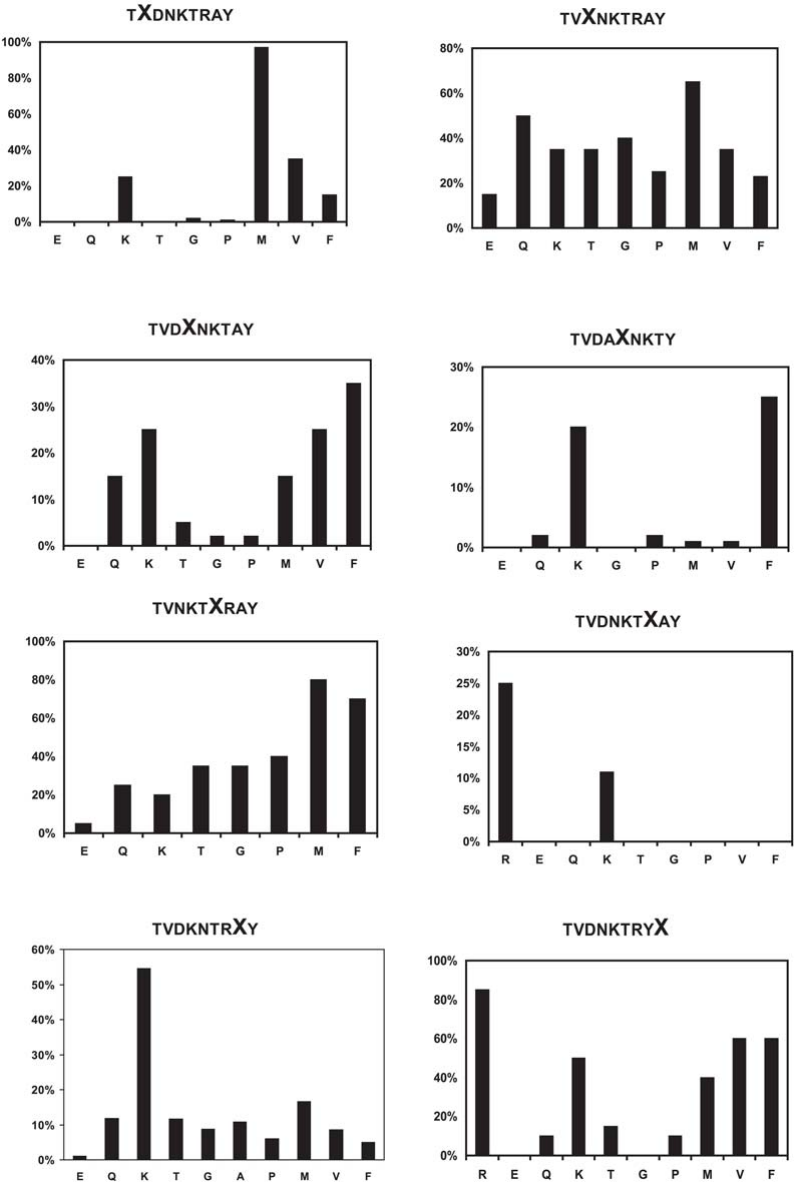
Trimming of the N-terminal residue of a library of 9mer peptides by ERAP1. Peptide series vary in one position per collection (indicated on the top of each panel; varying amino acid is shown as X) and are presented from left to right and from top to bottom for positions 2 to 9 from the N-terminus. The y-axis indicates percentage of substrate (peptide) depleted based on analysis by HPLC. The x-axis indicates the amino acid in the particular peptide in each collection, ordered by hydrophobicity (from hydrophilic amino-acids to hydrophobic). Note that for some collections the effect of substituting for particular amino acids is much higher than in others. One representative experiment is presented for each peptide set.

To systematically test the role of internal residues of peptide substrates on the rate of N-terminus trimming by ERAP1 we used the active recombinant enzyme in degradation assays with an already available collection of more than 70 synthetic model peptides (Figure 4). This peptide collection has been used before in the investigation of the specificity of the peptide transporter TAP [11]. All peptides were 9mers and had a threonine residue at their N-termini. Degradation of the N-terminus of each peptide was followed by HPLC as described in the experimental section. Each peptide series (varying at one position) was analyzed in parallel to account for variability in enzyme activity between preparations and during storage. Several experiments were performed for each peptide series to fine-tune the reaction conditions (reaction time and amount of enzyme used) and to test reproducibility of results. One representative set is shown in Figure 4. The efficiency of ERAP1 trimming was strongly affected by the nature of the residue at several positions in the peptide sequence (Figure 4). Specifically, positions 2, 5 and 7 (with position 1 defined as the N-terminal residue of the peptide) were found to be most important for the sensitivity of the peptide to ERAP1 degradation. Some degree of residue preference was also evident for positions 4, 8 and 9. Positions 3 and 6 showed the least specificity although some small effects were present. Residue preferences were only seen for hydrophobic and positively charged residues. No preference was seen for negative or hydrophilic residues in any of the positions. The presence of a negatively charged residue (glutamate) anywhere in the peptide sequence seemed to negatively affect the peptide's degradation by ERAP1 regardless of its location in the peptide sequence (to a lesser extent for position 3). The same general observation seems to apply for glycine and proline residues. Certain positions showed a very strong preference for particular amino-acid side-chains. Position 2 for example, exhibited a strong preference for a methionine residue whereas position 7 showed a very strong preference for positively charged residues (lysine or arginine). Interestingly, position 5 showed a strong preference for either a positive charge or an aromatic residue (phenylalanine). This “dual” preference was observed in other positions also (position 9) and may indicate alternative binding configurations for the two peptides (refer to the molecular modeling section below). Overall, strong sequence preferences were clearly evident from this library screen even without a more detailed kinetic study. To simplify screening, a single time point analysis was used in Figure 4 and as a result some of the differences seen there could be under-estimations of the kinetic differences, especially for reactions where the substrate consumption is over 50%. However, several of the preferences are so strong that are clearly evident even from a single time-point analysis.

**Figure 5:**
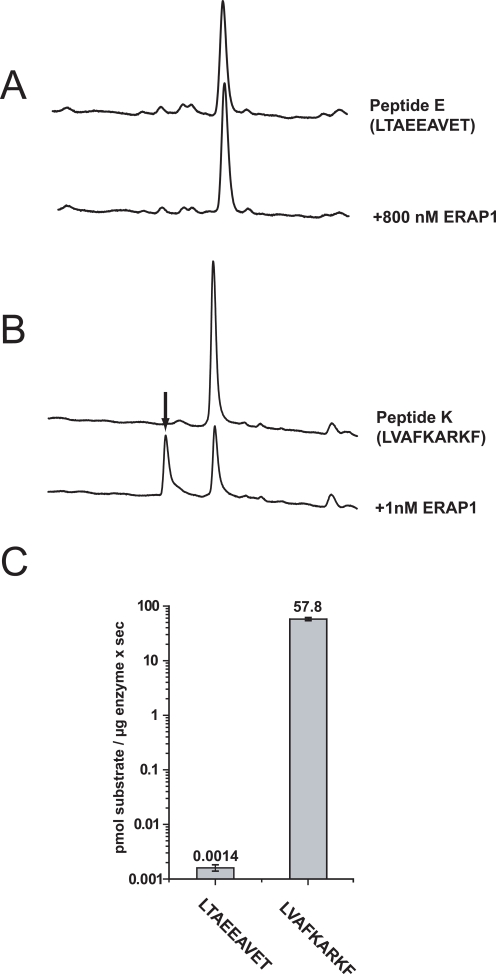
Validation of library screen. The most pronounced preferences found from the library screen in figure 4 were used to design two peptides that would be expected to be a bad and a good ERAP1 substrate respectively. Trimming of the N-terminal leucine of peptides E (sequence LTAEEAVET) and K (sequence LVAFKARKF) was followed by HPLC. The two peptides differ at 6 internal positions, carrying at those positions the amino acids that were found to be the least and the most preferred by ERAP1 respectively. *Panel A*, 50 µM peptide E was incubated with 800 nM ERAP1 for 1 hr at 37°C and the digestion products analyzed by reverse-phase chromatography. Peptide E was completely resistant to trimming under those conditions. *Panel B* 50 µM peptide K was incubated with 1 nM ERAP1 for 1 hr at 37°C and the digestion products analyzed by reverse-phase chromatography. Peptide K was efficiently trimmed (arrow points at product peak) under these conditions. *Panel C*, trimming rates for peptides E and K by ERAP1, average of 3–4 experiments. The y-axis is logarithmic for better visualization of the difference. ERAP1 trims peptide K more than 40,000 times faster than it trims peptide E.

To validate the library results we designed two model peptides based on the preferences observed in Figure 4. The trimming of the N-terminal leucine residue of peptide LVAFKARKF (peptide K) and LTAEEAVET (peptide E) by ERAP1 was evaluated by reverse-phase HPLC. Peptide K differs from peptide E at 6 positions, carrying amino acids that were found to be preferred by ERAP1 based on the 9mer library screen. In contrast peptide E, carries one of the least preferred amino acids for each of these positions. Both peptides have the same N-terminal residue, allowing the examination of preferences distal to the N-terminus of the peptide. Peptide E was highly resistant to trimming by ERAP1 even at µM enzyme concentrations whereas peptide K was trimmed efficiently even at nM enzyme concentrations (Figure 5, panels A and B). To investigate this further, we measured the trimming rate of the N-terminal residue of the two peptides by ERAP1. ERAP1 trimmed peptide K with a rate of 57.8±3.8 pmol/µg×sec and peptide E with a rate of 0.0014±0.0002 pmol/µg×sec a difference of more than 40,000 fold (Figure 5C). This impressive difference suggests that single positional side-chain preferences by ERAP1 can synergize to larger sequence preferences.

Our results for ERAP1 sequence preferences are in sharp contrast to the results obtained with the same library for TAP transport preferences where only the C-terminal residue was found to be important [11]. Such specificity effects originating from side-chains distal from the scissile N-terminal peptide bond are, to our knowledge and up to date, unique to this aminopeptidase and have only been partially observed before as differential substrate specificity depending on the C-terminus of an unrelated series of peptides [16]. Our results suggest that ERAP1 recognizes sequence (amino-acid side chains) in the peptide substrate it degrades. The ramifications of this finding to our understanding of antigenic peptide generation and destruction are discussed below.

### Homology modelling of ERAP1 based on the structure of TIFF3 reveals an extended putative peptide-binding site that has a strong negative electrostatic potential

**Figure 6:**
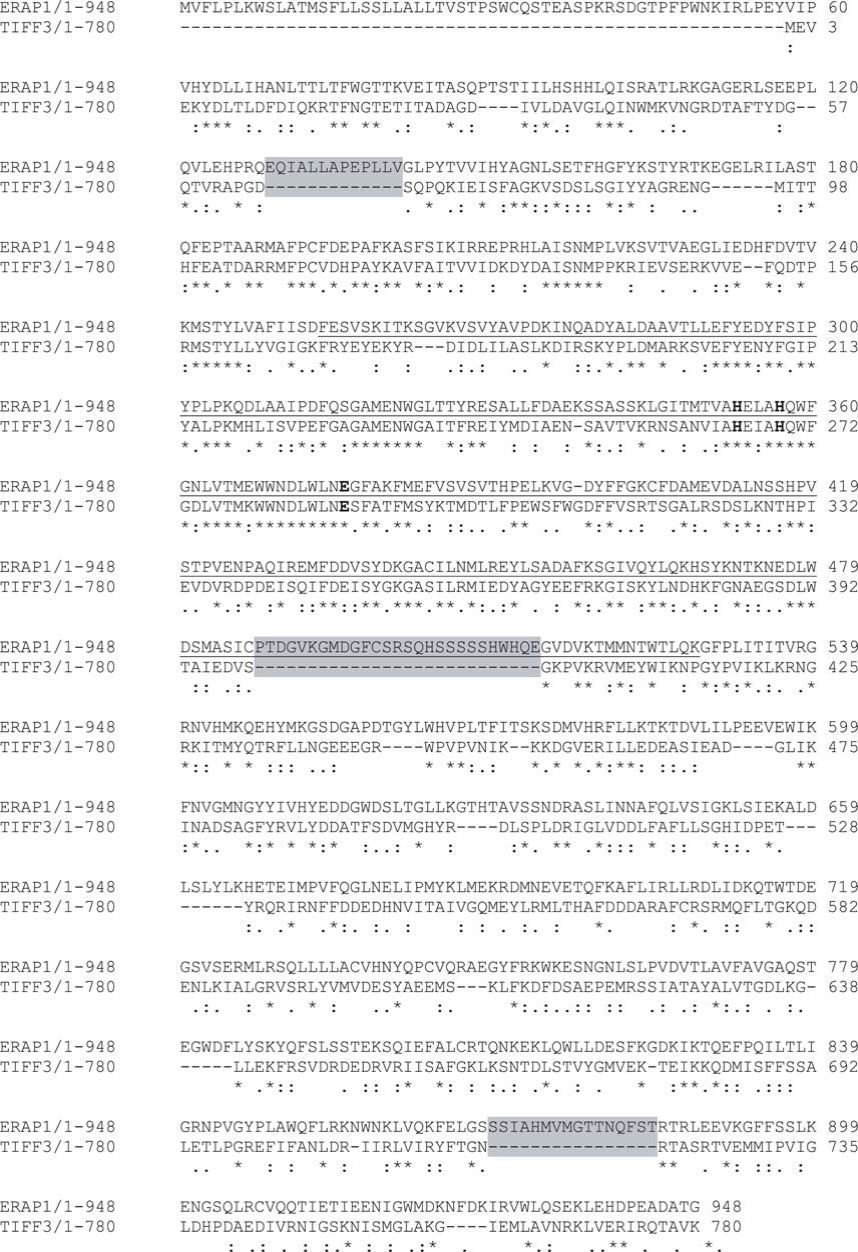
Sequence alignment of ERAP1 with Tricorn Interacting Factor F3 (TIFF3). The two proteins have a 28% sequence identity in the amino acid range of 58–948 and up to 43% sequence identity in the range of 280–486 that is the major part of the catalytic domain (underlined). Alignment reveals the presence of 3 major gaps (in grey).

To form an atomic level framework for understanding the sequence specificity effects observed during screening model peptides we constructed a homology model for ERAP1 based on the structure of the sequence related enzyme Tricorn Interacting Factor F3 (TIFF3). In the absence of a high-resolution structure of ERAP1, TIFF3 is the closest homolog to ERAP1 for which a high-resolution crystal structure is available [38]. By virtue of a BLAST homology search, ERAP1 displays 32% identity (residues 63–665) with TIFF3, 25% identity (residues 180–484) with the human leukotriene A4 hydrolase (LTA4H); and 25% identity (residues 180–489) with aminopeptidase N from Escherichia Coli (pepN). Full sequence alignment of ERAP1 and TIFF3 using ClustalW 1.83 exhibits 28% identity (residues 58–948) with three major gaps (grey regions). However, ERAP1_[280–486]_ that comprises the major part of the catalytic domain displays 43% sequence identity to the equivalent domain of TIFF3 with 84% identity at the core of the zinc-binding domain (residues 352–382). The alignment of ERAP1 with TIFF3 is shown in Figure 6.

**Figure 7:**
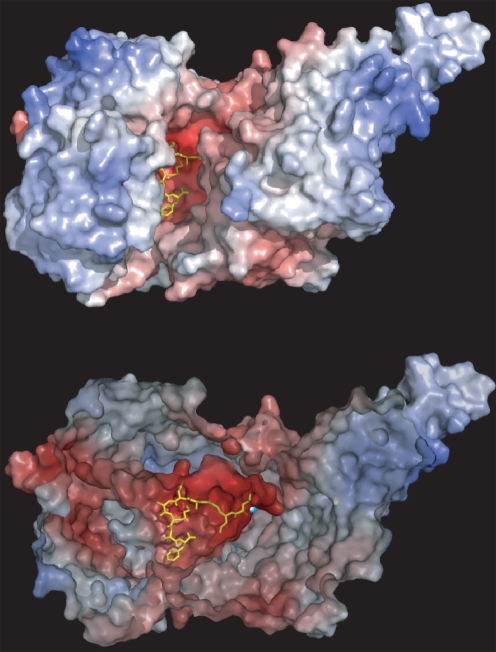
Homology model of ERAP1 based on the TIFF3 structure. *Top panel*: The model is displayed in surface representation and coloured by electrostatic potential (red indicates negative electrostatic potential, blue indicates positive electrostatic potential). Notice the highly negatively charged (red) wide groove in the middle of the molecule adjacent to the catalytic Zn(II). The model peptide (of the sequence LMAAFAKAF in yellow) designed based on the library results to represent a near optimal substrate was docked in the active site so that the scissile amino-terminal peptide bond is positioned adjacent to the catalytic site Zn(II). *Bottom panel*, detail view of docked peptide in postulated binding site; the obstructing part of the protein has been removed to allow easier visualization of the peptide. The predicted location of the catalytic Zn(II) is indicated as a cyan sphere. Electrostatic potentials were calculated by the PME electrostatics add-on of VMD 1.8.5 and picture created using Pymol 0.99.

The ERAP1 models we generated exhibit structural characteristics that appear highly relevant to the peptide library screen results. A central wide and deep cavity leads to the active site Zn(II) and is an obvious candidate to accommodate the peptide substrate (Figure 7, top panel). The cavity is large enough to accommodate a 9mer or even larger peptide (Figure 7, bottom panel). The cavity carries a strongly negative electrostatic potential but at the same time contains several hydrophobic pockets. The equivalent region of TIFF3 displays no such electrostatic potential distribution (data not shown). Based on the crystallographic analysis of TIFF3 there are 3 different molecular models that slightly vary in their inter-domain configuration. Depending on the TIFF3 model used for the construction of the ERAP1 homology model the opening to the aforementioned central cavity varies slightly in size; however the gross features (electrostatic potential and size) were found to be virtually unchanged (data not shown).

To gain insight on how peptide-substrates could interact with the enzyme we employed docking calculations of model peptides into the homology model of ERAP1. The model peptides were restricted only in that the scissile N-terminal bond had to be adjacent to the active site Zn(II) (Figure 7). In every case, the whole length of the model peptide was easily accommodated and was found to form an extended network of interactions with ERAP1's putative binding cavity. However, due to the relatively low homology between ERAP1 and TIFF3 that can lead to uncertainties in the ERAP1 model, the found interactions between the peptide and ERAP1 were not further evaluated. It should be noted though that interactions between the positively charged residues in the C-terminal moiety of the model peptides and negatively charged residues in ERAP1's putative binding cavity potentially explain the strong substrate specificity effect seen for positively charged side chains. Conversely, the negative electrostatic potential of the cavity would be expected to hinder the approach and binding of negatively charged peptides (especially with charges in the C-terminal moiety) an observation that could explain the negative effect of glutamate residues in peptide degradation. Overall, the ERAP1 homology model seems to provide an appropriate framework for at least a gross understanding of the basis of substrate specificity of ERAP1. A high-resolution structure of ERAP1 would be required for more reliable modelling calculations.

### Sequence effects on trimming of longer peptides carrying N- or C-terminal extensions of the same core sequence

**Figure 8:**
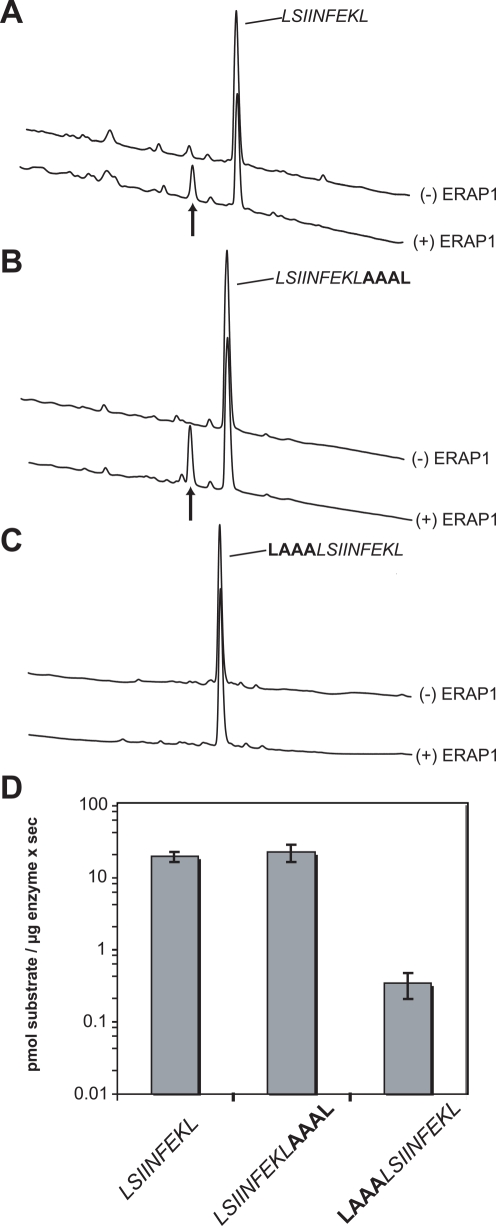
Trimming of the N-terminal leucine of N- and C- extended versions of LSIINFEKL peptide. *Panels* A–C, typical C18 chromatograms comparing the trimming for LSIINFEKL and the C- and N-terminal extended versions LSIINFEKLAAAL and LAAALSIINFEKL. In each panel two chromatograms are shown, the top one in the absence of enzyme and the bottom in the presence of 40 ng ERAP1. All incubations were done for 1 hr at 37°C. Black arrows indicate new peaks detected after incubation with the enzyme. Under these conditions LSIINFEKL and LSIINFEKLAAAL are trimmed about to the same extent and LAAALSIINFEKL is resistant to any trimming. *Panel* D, ERAP1 trimming rates calculated as the average of 3 to 5 experiments. Because of the large difference between trimming rates the y-axis is logarithmic for better visualization.

Many of the antigenic peptide precursors that are sent to the ER through the action of TAP are peptides up to 13–15 amino acid [39]. To test whether strong internal sequence related effects are also evident when ERAP1 is trimming peptides of such lengths and also to gain insight on the mode of molecular recognition of peptides by ERAP1 we measured the N-terminal trimming of 3 peptides based on the sequence of the ovalbumin antigenic epitope SIINFEKL (Figure 8). A control peptide with the sequence LSIINFEKL was used that we expected (based on previously published results on closely related sequences[19]) to be trimmed efficiently by ERAP1 leading to the removal of the N-terminal leucine residue. Two more peptides were tested: a peptide with the sequence LSIINFEKLAAAL and one with the sequence LAAALSIINFEKL, both based on the same internal template LSIINFEKL but with C- and N-terminal alanine extensions respectively. We designed the extensions to contain alanine residues since alanine lacks an extended side chain that may offer extra interactions with the binding site of the enzyme and complicate this analysis. Since it has been established that both the N- and C- termini of the peptide are important for determining the rate of trimming by ERAP1, we designed all 3 peptides to carry the same N and C terminal residue.

The peptide LSIINFEKL was trimmed efficiently by ERAP1 with a rate of 19.6±3.3 pmol/µg ERAP1×sec (Figure 8A). Similarly the peptide with sequence LSIINFEKLAAAL (a C-terminally extended version of LSIINFEKL) was trimmed just as efficiently with a rate of 22.4±5.8 pmol/µg ERAP1×sec (Figure 8B). In contrast, the peptide LAAALSIINFEKL (an N-terminally extended version of LSIINFEKL) was trimmed very poorly by ERAP1 (0.34±0.13 pmol/µg ERAP1×sec) and when incubated under identical conditions as the other two peptides, it was not trimmed at all (Figure 8C). Figure 8D shows a graphical representation of the trimming rates on a logarithmic scale for clarity. Notice how the trimming rate was essentially identical for LSIINFEKL and LSIINFEKLAAAL but about 100 fold smaller for LAAALSIINFEKL. These results clearly highlight how the internal sequence of the peptide can greatly affect N-terminus trimming rate by ERAP1. Furthermore, the similarity in trimming rates between LSIINFEKL and the C-terminally extended version LSIINFEKLAAAL, suggests that both substrates are recognized in a similar fashion by the enzyme and that ERAP1 substrate recognition occurs in reference to the N-terminus of the peptide.

### Generality of sequence preferences

**Figure 9:**
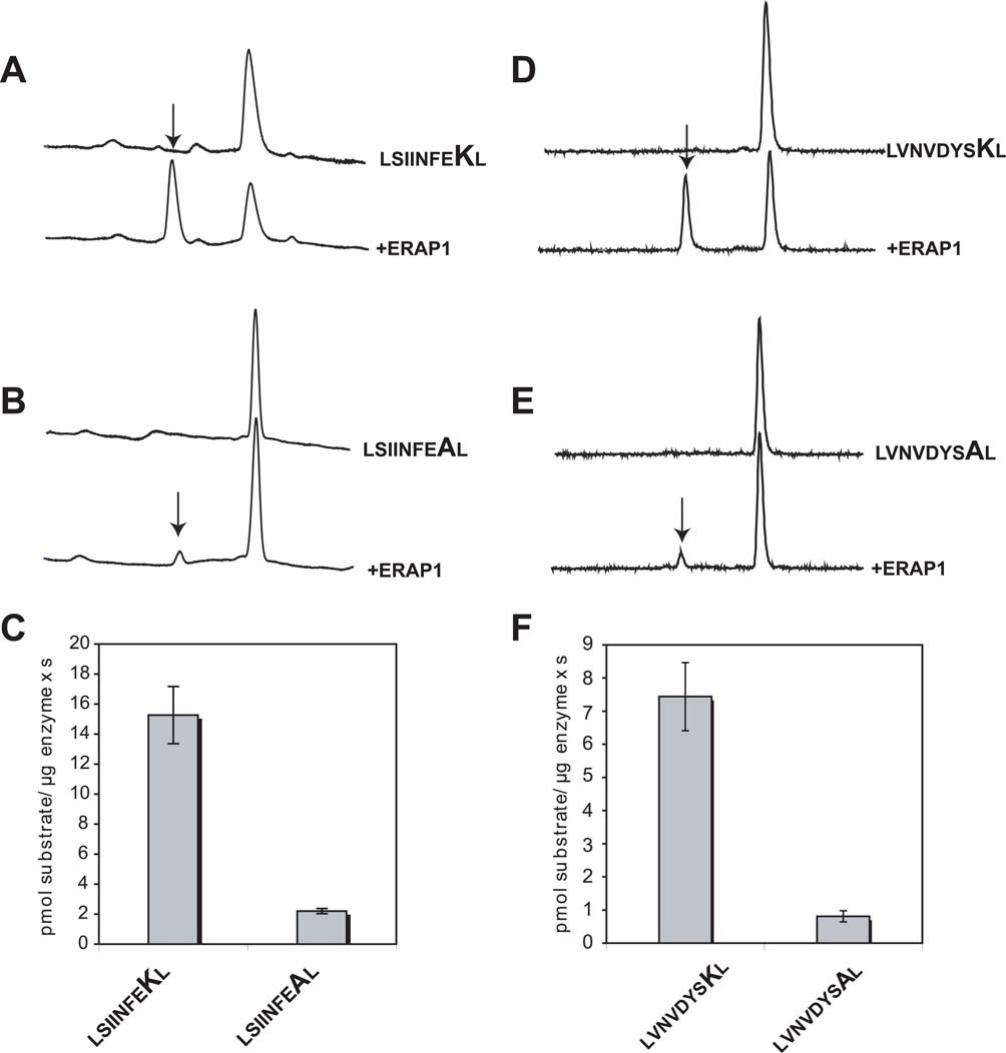
Substitution of a lysine residue at position 8 of two model 9mer H2-K^b^ epitope precursors leads to a marked decrease of N-terminus trimming by ERAP1 *in vitro*. *Panels* *A* *and* *B*, peptides LSIINFEKL and the variant LSIINFEAL (both at 50 µM) were incubated with 7.8 nM of ERAP1 for 1 hr at 37°C and the products analyzed by RP-HPLC. The product of the digestions, either SIINFEKL or SIINFEAL is marked on the chromatogram by an arrow. *Panel* *C*, trimming rates for excision of the N-terminus of peptide LSIINFEKL and LSIINFEAL by ERAP1, average of 3 separate experiments. *Panels* *D* *and* *E*, peptides LVNVDYSKL and the variant LVNVDYSAL (both at 50 µM) were incubated with 15.6 nM of ERAP1 for 1 hr at 37°C and the products analyzed by RP-HPLC. The product of the digestions, either VNVDYSKL or VNVDYSAL is marked on the chromatogram by an arrow. *Panel* *F*, trimming rates for excision of the N-terminus of peptides LVNVDYSKL and LVNVDYSAL by ERAP1, average of 3 separate experiments. Note that in both cases, substituting an alanine for the lysine at position 8, leads to a decrease of N-terminus trimming rate by about 8–10 fold.

Both the alanine scan in Figure 3 and the library screen in Figure 4 revealed a strong preference for a positively charged residue at position 8 relative to the N-terminus of the peptide. To test whether such a preference is more general we measured the trimming rate of two model antigenic peptide precursors based on epitopes of the MHCI molecule H-K^b^, namely LSIINFEKL and LVNVDYSKL (Figure 9). Both peptides carry identical N- and C- termini, as well as a lysine residue at position 8 relative to the N-terminus. Both peptides were efficiently trimmed by ERAP1, resulting to the mature antigenic epitope. In both cases substitution of the position 8 lysine residue by an alanine lead to a marked decrease in trimming rate by ERAP1 by 8–10 fold (Figure 9). This rate change is consistent with the decrease in trimming for the series TVDKNTRXY in Figure 4 when a lysine is compared to an alanine. Furthermore, it is consistent with the effect seen for the 10mer in Figure 3, although the change in rate was more pronounced in that case. Overall, we demonstrate that eradication of a positive charge at position 8 of four unrelated peptide sequences leads to a marked reduction in trimming by ERAP1, suggesting that this effect might be more general.

## Discussion

In this study we used collections of model peptides to systematically study the effects of peptide sequence in the degradation of its N-terminus by ERAP1. It should be noted that previous work with other aminopeptidases has indicated strong preferences only for the N-terminal residue of the peptide as well as faster trimming for smaller peptides [16]. In contrast, ERAP1 trims long peptides better than short ones and its trimming rate can be also affected by the nature of the C-terminus of the peptide [16]. We find here that the unique properties of ERAP1 extend to strong internal sequence specificity requirements for peptide substrate trimming. Negatively charged side-chains on the peptide seem to make it a worse substrate for ERAP1 as would be predicted from the negative electrostatic potential of ERAP1's putative peptide-binding site (Figure 7). In contrast, we find strong preference for positively charged and hydrophobic residues for several positions in the peptide-substrate. These effects, taken together, suggest an extended molecular recognition throughout the whole length of the peptide by ERAP1.

### Molecular mechanism of ERAP1 peptide trimming

The striking properties of ERAP1 compared to known aminopeptidases (length and sequence specificity distal to the scissile bond) can only be understood in terms of unique structural features of this enzyme. In the absence of a high-resolution crystal structure we constructed a homology model using the structure of the related aminopeptidase TIFF3. Although the model is of limited use for any high-resolution structural conclusions, we anticipate that at least the gross structural features of the protein will be reasonably accurate. Analysis of the electrostatic potential distribution of the ERAP1 model revealed a wide central cavity with a strong negative potential. This cavity is large enough to accommodate model peptides in orientations that position their N-terminal scissile peptide bond in close proximity to the catalytic Zn(II) atom (Figure 7). The electrostatic potential of the cavity can easily explain why model peptides that carry negative charges are poorer substrates for the enzyme as well as the preference for positive charges at several positions. Docking calculations with model peptides yielded low energy conformations with several potential side chain interactions between the peptide side-chains and amino acids in the cavity of ERAP1. It is conceivable that interactions between such a presumed cavity and peptide substrates are responsible for the peptide specificity effects of ERAP1. Additionally, this cavity is large enough to accommodate even larger peptides (particularly since the C-terminus of the peptide can extend outwards towards the solvent) with their N-terminus located adjacent to the catalytic Zn^++^. This mode of recognition is consistent with our results that demonstrated similar trimming kinetics between a model peptide and its C-terminal extended version and very different trimming kinetics between the peptide and its N-terminal extended version (Figure 8). We therefore suggest that the substrate preferences we demonstrate in this study arise from specific molecular interactions between the peptide and an extended peptide binding site that lies adjacent to ERAP1's Zn(II) catalytic site.

### Generality of trimming preferences

Since all enzymatic activity comparisons were performed within each peptide collection our results are most readily generalized in that context; i.e. substitution of a positively charged amino acid (like lysine) at position 8 for an uncharged one (like alanine) would be predicted to reduce N-terminal trimming rate by ERAP1 and vice versa. The generality of our observations is supported by the fact that we find internal sequence effects in several unrelated peptide sequences of different lengths (10mer in Figure 3, 9mers in Figure 4, 13mers in Figure 8 and 9mers in Figure 9). In addition, the strong effect seen in N-terminus trimming when the positively charged residue at position 8 of the peptide LYWANATRSG (Figure 3) is substituted by alanine is consistent with the preference for positively charged residues at the same position in the peptide series TVDKNTRXY (Figure 4). The same effect is seen when the single positively charged residue is substituted by alanine in the model precursors of natural occurring antigenic epitopes LSIINFEKL and LVNVDYSKL (Figure 9), suggesting that this preference for positive charges might be more general. Furthermore, the sequence LSIINFEKLAAAL compared to the sequence LAAALSIINFEKL would be predicted to be preferred due to residues at positions 6 (phenylalanine versus serine; phenylalanine is found to be preferred over the serine homolog threonine in the library in Figure 4), position 8 (lysine versus isoleucine, lysine is preferred over the isoleucine homolog valine in the library) and position 9 (leucine versus asparagine; the leucine homolog valine is preferred over the asparagine homolog glutamine in the library). Due to the limited residues tested in the library no clear conclusion can be reached for the remaining positions. However, every single position for which a conclusion can be reached, favors LSIINFEKLAAAL over LAAALSIINFEKL. Possible synergy between the preferred residues in LSIINFEKLAAAL can potentially explain the 100-fold faster trimming rate. Whether the cumulative specificity effect from more than one position in the peptide is additive or synergistic is a potentially important question for substrate prediction that requires further experimentation to answer. In any case, the strong internal sequence specificity effects observed in our study, in combination with the structural features of the enzyme's peptide-binding site (i.e. negative electrostatic potential, Figure 7), suggest that the ERAP1 trimming preferences are not restricted to the limited sequence templates tested here, but might be more general. However, further analysis with peptide collections based on multiple unrelated sequence templates may be necessary to establish clear substrate sequence recognition motifs.

### Antigenic peptide generation and ERAP1 substrate preferences

Several publications during the last few years have highlighted the importance of ERAP1 in generating peptides for antigen presentation. Its sub-cellular localization, induction by interferon-γ and peptide substrate length preference all fit well with a specific role in preparing peptides before loading onto nascent MHC class I molecules [14]–[16], [19]. Furthermore, antigenic peptide presentation assays have indicated that ERAP1 can have complex effects on antigenic epitopes depending on the nature of the epitope tested [14], [19]. Recent work by four independent laboratories has examined the effect of deletion of the ERAP1 gene in mice and concluded strong, epitope-dependent effects in antigenic presentation [24]–[26], [40]. Generally the effect of ERAP1 activity in vivo seems to be complex; down-regulation or over-expression of ERAP1 levels have different and often opposite results for different epitopes, as well as negligible results for others. One factor that differentiates between epitopes is their sequence as well as the sequence of their extended precursors. If ERAP1, as suggested by our data, trims peptides at dramatically different rates depending on their internal sequence and this also applies inside the cell, it would not be surprising that ERAP1 down-regulation would lead to different effects depending on the epitope studied. Furthermore, most MHCI epitopes are 9mers and we find that ERAP1 can trim about half of the 9mers tested in Figure 4, depending on sequence. Such an activity would effectively destroy the antigenic epitope, again in a sequence dependent manner. Down-regulation of ERAP1 activity would therefore be expected to enhance the abundance of such epitopes. Overall, our findings suggest that it is possible that ERAP1's activity poses a bias in the generated sequence of antigenic peptides by trimming antigenic peptide precursors at very different rates depending on the precursor sequence. It is not inconceivable that this bias can be related to the editing properties attributed to the enzyme [27], [41]. Although more detailed work is needed to fully understand this effect, it is possible that the unique substrate specificity of ERAP1 is one of the reasons behind the complex effects seen with epitopes of different sequences in deletion experiments.

In summary, in this study we have characterized the substrate specificity of ERAP1, an important enzyme in the generation of antigenic peptides that has received a great deal of attention by the scientific community the last few years. Our results demonstrate that ERAP1 has strong internal sequence preferences for the peptide-substrate that can potentially strongly affect peptide trimming rates. These preferences are possibly mediated by a unique for this aminopeptidase, extended peptide binding site. We therefore propose that the whole sequence and not just the N-terminus or the C-terminus of the antigenic peptide precursor, is the determining factor in the efficiency of generation or even destruction of an antigenic peptide by ERAP1.
